# Facteurs associés à la létalité chez les patients hospitalisés pour le VIH avancé

**DOI:** 10.5588/pha.23.0009

**Published:** 2023-08-01

**Authors:** D. Abdourahimi, D. Yehadji, E. Briskin, E. M. Khine, C. Arias, K. S. André, F. K. Mukebela, L. Ndayisenga, P. Isaakidis, E. C. Casas, S. J. Steele, F. B. Sacko, G. Foromo

**Affiliations:** 1 Médecins Sans Frontières (MSF), Conakry, Guinée; 2 Médecins Sans Frontières, Luxembourg Operational Research (LuxOR) unit, Luxembourg City, Luxembourg; 3 MSF, South African Medical Unit (SAMU), Le Cap, Afrique du Sud; 4 Hôpital National de Donka, Conakry, Guinée; 5 Programme National de Lutte contre le VIH et les Hépatites (PNLSH), Conakry, Guinée

**Keywords:** virus de l’immunodéficience humaine, syndrome de l’immunodéficience acquise, décès, mortalité

## Abstract

**CONTEXTE:**

Une unité soutenue par Médecins Sans Frontières (MSF) qui prend en charge les patients avec un VIH avancé à l’Hôpital National de Donka, Conakry, Guinée.

**OBJECTIF:**

Déterminer les facteurs liés à la survenue du décès chez les patients hospitalisés dans l’unité entre 2017 et 2021.

**SCHÉMA:**

Ceci est une analyse rétrospective de données de routine des patients hospitalisés pour le VIH avancé.

**RÉSULTATS:**

Au total, 3,718 patients étaient inclus, d’âge médian de 40 ans (IQR 33–51), dont 2,241 (60,3%) femmes. Le taux de moyen de décès était de 33,6% (*n* = 1,240). Il était passé de 40% en 2017 à 29% en 2021, sans être statistiquement significatif. La période la plus à risque de décès était les 25 premiers jours d’hospitalisation. Chez ces patients décédés, la TB (43,8%) et la toxoplasmose (11,4%) étaient les diagnostics les plus fréquents. Après analyse multivariée par régression de Cox, les facteurs associés au décès étaient un âge compris entre 25–49 ans (*hazard ratio* ajusté [HRa] 1,60 ; *P* = 0,002) ou ⩾50 ans (HRa 1,80 ; *P* < 0,001), la présence de signes respiratoires (HRa 1,23 ; *P* = 0,001) ou abdominaux (HRa 1,26 ; *P* < 0,001) et une réadmission (HRa 0,54 ; *P* < 0,001).

**CONCLUSION:**

Les patients âgés de 25–49 ans, ou plus, ou présentant des signes respiratoires ou abdominaux requièrent une surveillance accrue car ils sont les plus à risque de décéder de la maladie, surtout pendant les 25 premiers jours d’hospitalisation.

## Abstract (en)

**CONTEXT:**

A unit supported by Médecins Sans Frontières (MSF) cares for patients with advanced HIV at Donka National Hospital, Conakry, Guinea.

**OBJECTIVE:**

To determine the factors associated with the occurrence of death in patients hospitalised in the unit between 2017 and 2021.

**DESIGN:**

This was a retrospective analysis of routine data from patients hospitalised with advanced HIV.

**RESULTS:**

A total of 3,718 patients were included, with a median age of 40 years (IQR 33–51), of whom 2,241 (60.3%) were women. The mean mortality rate was 33.6% (*n* = 1,240), down from 40% in 2017 to 29% in 2021, but this was not statistically significant. The period most at risk of death was the first 25 days of hospitalisation. Among these patients, TB (43.8%) and toxoplasmosis (11.4%) were the most frequent diagnoses. After multivariate analysis using Cox regression, the factors associated with death were age 25–49 years (adjusted hazard ratio [aHR] 1.60; *P* = 0.002) or ≥50 years (aHR 1.80; *P* < 0.001), the presence of respiratory (aHR 1.23; *P* = 0.001) or abdominal symptoms (aHR 1.26; *P* < 0.001) and readmission (aHR 0.54; *P* < 0.001).

**CONCLUSION:**

Patients aged 25–49 years or older, or those presenting with respiratory or abdominal signs require increased surveillance, as they are at the greatest risk of dying from the disease, especially during the first 25 days of hospitalisation.

Après plus de trois décennies, malgré la disponibilité des traitements antirétroviraux (ARV) efficaces, le VIH demeure une préoccupation majeure de santé publique, avec 38,4 millions de personnes vivant avec le VIH en 2021.^1^ Après une réduction notable de la mortalité avec l’application de la stratégie « dépister et traiter », elle semble dernièrement stagner. En 2021, à l’échelle mondiale près de 650 000 personnes sont décédées de maladies liées au syndrome d’immunodéficience humaine acquise (SIDA), soit à peine 4% de moins que l’année précédente.^1^

Pour réduire considérablement cette mortalité, il est nécessaire de planifier, financer et mettre en œuvre un paquet de soins afin de prévenir, détecter et traiter le VIH avancé ou le syndrome d’immunodéficience acquise, comme indiqué dans les lignes directrices de l’OMS en 2017.^2,3^ Un rapport initié par le Consortium sur le VIH avancé indique qu’un nombre croissant de pays à revenus faibles ou intermédiaires, s’intéressent au VIH avancé dans leurs directives nationales ; mais les difficultés de financement et de mise en œuvre d’un paquet de soins réduit l’effectivité sur le terrain.^2^

En Guinée, la prévalence est relativement faible, environ 1,5% de la population du pays selon le rapport de l’ONUSIDA en 2021.^1^ Cependant, plusieurs facteurs (la stigmatisation, le manque de ressources, etc.) rendent difficile et peu efficace la prise en charge, avec actuellement 52% de couverture en ARV.^4^ Le pays dispose néanmoins d’un Programme National de Lutte contre le VIH et les Hépatites (PNLSH), et des sites de prise en charge du VIH sur toute l’étendue du territoire.

Dans plusieurs hôpitaux, de nombreux patients naïfs d’ARV ou prétraités, se présentent ou reviennent aux soins après une longue période d’interruption, dans un état de VIH avancé, avec une mortalité élevée.^2^ Plusieurs travaux ont démontré le risque élevé de décès chez ces patients.^5,6^ Une étude menée à l’Hôpital de Kisangani en République Démocratique du Congo rapporte une mortalité de 25%.^5,7^ Cette létalité est sans doute influencée par certains facteurs, dont la fréquence et l’importance sont très variables selon les caractéristiques de la région, telles que socio-économiques, biologiques, culturelles, infrastructurelles et organisationnelles.

En Guinée, les facteurs liés au VIH avancé ne sont pas encore suffisamment documentés. De plus, ces dernières années, la politique de prise en charge des patients souffrant du VIH avancé a connu certaines modifications en accord avec les recommandations de l’OMS publiées en 2017.^8^ En effet les personnes vivant avec le VIH (PVVIH) nouvellement dépistés bénéficient d’un dosage des lymphocytes CD4 ; et ceux parmi eux souffrant du VIH avancé, c’est-à-dire, un taux de lymphocytes T CD4 < 200 cellules/µl ou à un Stade 3 ou 4 de la classification de l’OMS, bénéficient d’une recherche systématique des deux infections opportunistes sévères, à savoir la TB et la cryptococcose, respectivement par le test liporabinomannane (TB-LAM) et l’antigène cryptococcal (CrAg), afin d’améliorer leur survie.^2,9^

Le présent travail initié dans un tel contexte vise à identifier les facteurs associés à la létalité chez les patients souffrant du VIH avancé dans le pays afin de prendre des mesures correctrices y afférents.

## MÉTHODOLOGIE

### Type d’étude

Il s’agit d’une analyse rétrospective de données de routine des patients hospitalisés à l’Hôpital National de Donka, Guinée, entre le 1^er^ janvier 2017 et le 31 décembre 2021.

### Cadre de l’étude

L’étude a lieu à l’Hôpital National de Donka, l’une des deux structures de niveau tertiaire en charge du suivi des PVVIH ayant développé des complications graves nécessitant des soins soutenus. MSF apporte depuis 2016 un support au PNLSH dans la prise en charge du VIH avancé à travers une unité d’hospitalisation spécialisée au sein de l’hôpital. Cette unité accueille environ 700 patients avec un VIH avancé chaque année. Ils proviennent essentiellement des structures appuyées par MSF, mais aussi d’autres structures sanitaires de la capitale ou autres communes du pays.

Cette unité etait composée de 31 lits, trois médecins et d’autres ressources diverses (infirmiers, hygiénistes, brancardiers, pharmacien, etc.). Les examens étaient réalisés par le laboratoire de Matam, Guinée. Différents changements sont intervenus courant ses 5 années de 2017, à savoir, l’unité possède 32 lits, dont quatre pour les soins intensifs et la réalisation des examens se fait soit à Matam, soit à l’unité de prise en charge ambulatoire de l’Hôpital National de Donka ou dans un laboratoire privé, pour, entre autres, la réalisation des examens requis à l’admission tels que le dosage des CD4, TB-LAM, et CrAg. Les ressources humaines y exerçant comprennent entre autres huit médecins, 18 infirmiers, une nutritionniste, un pharmacien, des psychologues et des techniciens de laboratoire. Les consultations sont réalisées par un médecin qui décide de son orientation vers les soins intensifs ou généraux. Les patients admis dans l’unité sont prélevés pour les bilans sus-énumérés. Le diagnostic posé par le médecin est validé par le superviseur médical des activités médicales. Les soins sont entièrement gratuits. Les patients qui nécessitent des soins spécialisés non disponibles dans l’unité sont référés vers des structures appropriées. Un accent particulier est mis sur la TB avec plusieurs molécules en cas de besoin pour alternative, le traitement de la cryptococcose est plus court, un volet de prise en charge de la malnutrition existe. Le décès est constaté par le médecin et les informations de la sortie sont notifiés dans le dossier du malade.

### Participants à l’étude

La population d’étude est composée de tous les patients admis dans l’unité pour le VIH avancé pendant la période de l’étude.

### Collecte des variables

Des informations sur les caractéristiques démographiques, cliniques, virologiques, les résultats de la numération des CD4, des examens TB-LAM et CrAg, le devenir ont été collectées à partir de la base de données de suivi de routine des patients admis. Elles ont été fusionnées avec une base de données provenant du logiciel de suivi des patients en ambulatoire pour constituer la base de données finale de l’étude. Cette dernière a fait l’objet d’une procédure de contrôle qualité des données pour vérifier la complétude et l’exactitude des données capturées, en se basant sur les outils primaires de collecte tels les dossiers patients et les registres.

### Analyses statistiques

Les données ont été analysées avec des logiciels Epi Info v7.2 et R v4.2 (R Computing, Vienna, Austria). Les variables quantitatives ont été décrites en utilisant les médianes (intervalle interquartile [IQR]), les variables qualitatives, les fréquences et les pourcentages. Afin de déterminer les périodes critiques, une courbe d’analyse de survie qui décrit la mortalité dans le temps a été reproduite en utilisant la méthode de Kaplan–Meyer. Les facteurs associés à la mortalité ont été déterminés par une régression de Cox. L’analyse bivariée a permis de déterminer le *hazard ratio *(HR) brut, son intervalle de confiance à 95% (IC 95%) et la valeur* P*. Tous les facteurs avec une *P* < 0,2 ont été inclus dans le modèle de régression multivariée. Le HR ajusté (HRa), son IC 95% et la valeur *P* ont été déterminés. Le seuil de significativité est fixé à 5%.

### Considerations ethiques

Cette recherche remplissait les critères d’exemption fixés par le Comité d’éthique de Médecins Sans Frontières, Génève, Suisse, pour les analyses a posteriori des données cliniques collectées de manière routinière et ne nécessitait donc pas d’examen MSF Ethics Review Board. L’approbation du Comité National d’Ethique pour la Recherche en Santé (CNERS), Conakry, Guinée a été obtenue (N° : 182/CNERS/22).

## RÉSULTATS

Au total, 3,718 patients, dont 2,241 (60,3%) femmes, ont été admis entre 2017 et 2021. Leur âge médian était de 40 ans (IQR 33–51). Parmi eux, 2,969 (79,8%) étaient nouvellement admis et 749 (20,2%) étaient réadmis. Les signes respiratoires et de malnutrition étaient présents respectivement chez 2,128 (57,2%) et 1,872 (50,3%) ; 2,586 (69,6%) patients avaient un taux de CD4 ≤200 cellules/µl ; et 926 (24,9%) n’avaient jamais été antérieurement sous ARV (Tableau 1).

**TABLEAU 1:** Caractéristiques cliniques, biologiques et statut vis-à-vis des antirétroviraux des patients admis à l’Hôpital National de Donka (Conakry), 2017–2021

	*n* (%)
Caractéristiques démographiques	
Sexe	
Hommes	1,477 (39,7)
Femmes	2,241 (60,3)
Age, ans	
⩾50	705 (19.0)
25–49	2,388 (64,4)
15–24	365 (9,8)
0–14	250 (6,7)
Centre de suivi	
Matam	1,145 (30,8)
Flamboyant	321 (8,6)
Gbéssia port	303 (8,2)
Wanidara	213 (5,7)
Minière	211 (5,7)
Tombolia	195 (5,2)
Coléah	135 (3,6)
Dabompa	54 (1,5)
Autre Conakry	49 (1,3)
Autre	1,092 (29,4)
Caractéristiques cliniques	
Signes respiratoires	2,128 (57,2)
Signes de malnutrition modérée ou sévère	1,872 (50,3)
Signes cutanés	1,433 (38,5)
Signes abdominaux	834 (22,4)
Signes méningés	680 (18,3)
Signes neurologiques	535 (14,4)
Taux de CD4, cellules/µl	
<100	1,941 (52,2)
100–200	645 (17,4)
>200	803 (21,6)
Non mesuré	329 (8,8)
Charge virale, copies/ml	
<1,000	697 (18,7)
1,000–50,000	533 (14,3)
>50,000	1,006 (27,1)
Non mesuré	1,482 (39,9)
Statut vis-à-vis des ARV	
Jamais sous ARV	926 (24,9)
Sous ARV<6 mois	846 (22,8)
Sous ARV⩾6 mois	1,324 (35,6)
Inconnu	622 (16,7)
Total	3,718 (100)

ARV = antirétroviraux.

**FIGURE 1:**
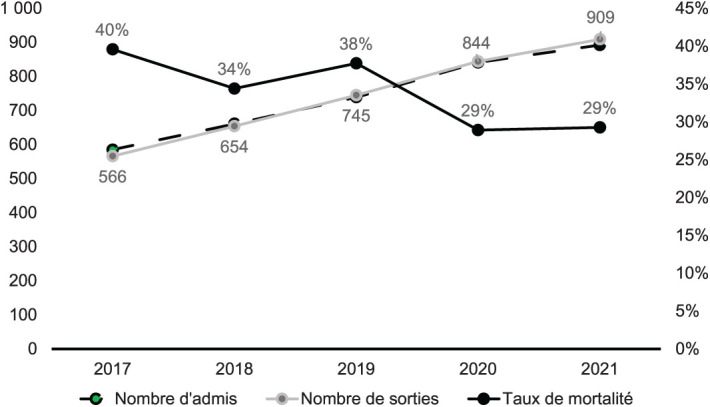
Evolution des admissions, des sorties et des décès chez les patients admis à l’Hôpital National de Donka (Conakry) de 2017 à 2021.

Les admissions ont connu une progression moyenne de 11% jusqu’en 2020, puis de 6% entre 2020 et 2021. La courbe des patients à l’exéat avait une allure similaire à celle des admission (Figure 1). Après les soins, 2,172 (58,9%) patients s’étaient améliorés et étaient rentrés à domicile, 1,240 (33,6%) étaient décédés, 243 (6,6%) étaient référés et 35 (0,9%) étaient rentrés contre avis médical. De 2017 à 2021, la mortalité était passée de 40% à 29%. Le taux de CD4 médian des patients admis s’est amélioré pendant ces 5 ans (*P* = 0,0103) (Figure 3).

**FIGURE 2:**
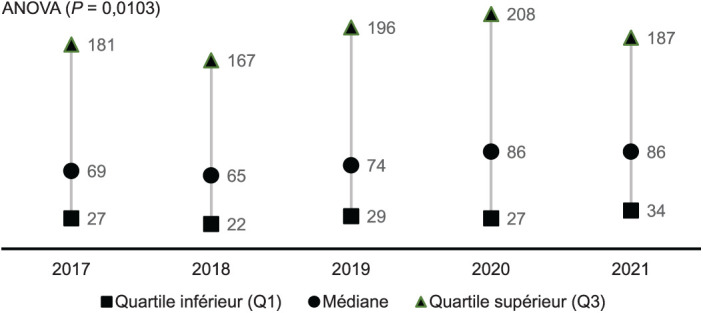
Evolution du taux de CD4 médian (IQR) chez les patients admis à l’Hôpital National de Donka, Conakry, Guinée, de 2017 à 2021. ANOVA = analyse de variance.

**FIGURE 3:**
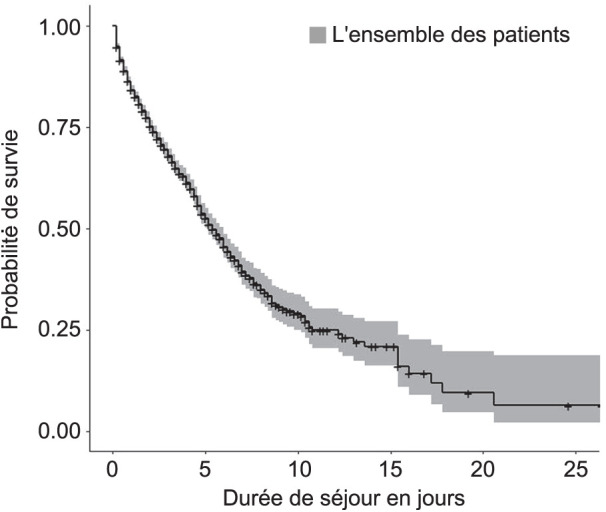
Courbe de survie chez les patients admis à l’Hôpital ­National de Donka, Conakry, Guinée, de 2017 à 2021.

**FIGURE 4:**
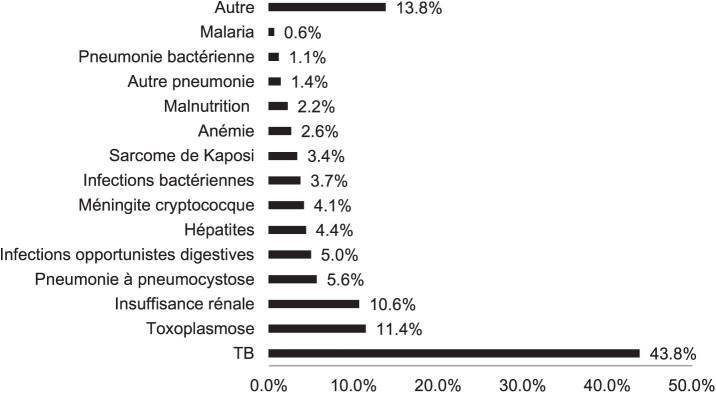
Diagnostics de sorties chez les patients décédés à l’Hôpital National de Donka, Conakry, Guinée, de 2017 à 2021.

D’après la courbe de survie (Kaplan–Meyer), le décès était surtout survenu pendant les 25 premiers jours d’hospitalisation (Figure 3). Après cette période, le risque de décéder était considérablement réduit. Le premier diagnostic de sortie retenu chez ces patients était la TB (43,8%), suivi de la toxoplasmose (11,4%) (Figure 4).

Après analyse multivariée, les facteurs associés à un risque élevé de décès étaient un âge compris entre 25–49 ans (HRa 1,60 ; *P* = 0,002) ou ≥50 ans (HRa 1,80 ; *P* < 0,001), la présence de signes respiratoires (HRa 1,23 ; *P* = 0,001) ou abdominaux (HRa 1,26 ; *P* < 0,001). Par contre, une réadmission était associée à un moindre risque de décès comparé aux nouvelles admissions, après ajustement sur les autres facteurs (HRa 0,54 ; *P* < 0,001) (Tableau 2).

**TABLEAU 2:** Facteurs associés aux décès chez les patients admis à l’Hôpital National de Donka, Conakry, pendant la période 2017 à 2021

	Total*n*	Décès *n* (%)	HRb (IC à 95%)	*P*-value	HRa (IC à 95%)	*P*-value
Sexe						
Hommes	1,477	516 (39,9)	1,12 (0,99–1,25)	0,061		
Femmes	2,241	724 (32,3)	1			
Age, ans				<0,001		
⩾50	705	264 (37,5)	1,71 (1,26–2,32)		1,34 (0,95–1,88)	0,095
25–49	2,388	822 (34,4)	1,52 (1,14–2,02)		1,60 (1,19–2,11)	0,002
15–24	365	99 (27,1)	1,19 (0,85–1,67)		1,80 (1,33–2,44)	<0,001
0–14	250	50 (20,0)	1		1	
Réadmission						
Oui	749	158 (21,1)	0,53 (0,45–0,63)	<0,001	0,54 (0,46–0,64)	<0,001
Non	2,969	1,082 (36,4)	1		1	
Malnutrition						
Oui	1,872	645 (34,5)	1,04 (0,93–1,17)	0,456		
Non	1,534	484 (31,5)	1			
Examen TB-LAM				0,477		
Pas fait	776	283 (36,5)	1,29 (1,11–1,03)	0,001		
Positif	1,368	467 (34,1)	1,03 (0,91–1,17)	0,582		
Négatif	1,574	490 (31,1)	1			
CrAg						
Positif	118	41 (34,8)	0,81 (0,59–1,09)	0,169		
Négatif	3,600	1,199 (33,3)	1			
<0,001						
Inconnu	329	169 (51,4)	2,86 (2,31–3,52)	<0,001		
100–200	645	190 (29,5)	1,09 (0,89–1,34)	0,409		
CD4<100	1,941	699 (36,0)	1,31 (1,11–1,54)	0,001		
CD4⩾200	803	182 (22,7)	1			
0,706						
Inconnu	1,482	612 (41,3)	2,11 (1,79–2,48)	<0,001		
⩾1,000	1,539	440 (28,6)	1,03 (0,87–1,23)	0,702		
<1,000	697	188 (27,0)	1			
Profil ARV				0,373		
Jamais ARV	926	303 (32,7)	1,06 (0,93–1,21)	0,366		
Inconnu	622	230 (37,0)	1,35 (1,16–1,57)	<0,001		
Sous ARV	2,170	707 (32,6)	1			
Signes cliniques à l’admission						
Cutanés						
Oui	1,433	503 (42,5)	1,07 (0,96–1,20)	0,2099		
Non	2,100	680 (57,5)	1			
Respiratoires						
Oui	2,128	767 (64,5)	1,24 (1,10–1,40)	0,0003	1,13 (1,09–1,38)	0,0004
Non	1,438	423 (35,6)	1			
Neurologiques						
Oui	535	219 (18,8)	1,13 (0,97–1,30)	0,1114		
Non	2,987	948 (81,2)	1			
Abdominaux						
Oui	834	346 (29,7)	1,27 (1,13–1,44)	0,0001	1,26 (1,10–1,42)	0,0004
Non	2,676	818 (70,3)				
Méningés						
Oui	680	286 (24,7)	1,16 (1,01–1,32)	0,0250		
Non	2,836	872 (75,3)				

HRb = *hazard ratio* brut ; IC = intervalle de confiance ; HRa = *hazard ratio* ajusté ; TB-LAM = liporabinomannane ; CrAg = antigène cryptococcal ; ARV = antirétroviraux.

## DISCUSSION

Ce travail qui avait pour but d’identifier les facteurs liés aux décès chez les patients avec le VIH avancé dans le pays nous montre que près de huit patients sur 10 étaient nouvellement admis dans un tableau de VIH avancé. Ce résultat interpelle et plaide pour une intensification de la sensibilisation sur le VIH dans la communauté et sa démystification, car de nombreux patients par crainte de stigmatisation cachent encore leur séropositivité, ne se présentant aux soins qu’à un stade très avancé de la maladie. Cependant l’implémentation du paquet CD4, TB-LAM et CrAg aussi pour les nouveaux patients dépistés en ambulatoire afin d’identifier rapidement le VIH avancé devrait être effective. L’immunodépression sévère, avec un taux de CD4 <200 cellules/µl retrouvée chez sept patients sur 10, l’absence totale de traitement ARV chez un quart d’entre eux, renforcent davantage la nécessité de mettre en œuvre des stratégies innovantes pour une sensibilisation plus efficiente de la communauté, en tenant compte de ses caractéristiques socio-culturelles et cultuelles spécifiques pour une meilleure efficacité.

Ensuite, le nombre d’admission a graduellement augmenté, bien qu’ayant connu un léger ralentissement entre 2020 et 2021, ce qui pourrait s’expliquer par l’apparition de la COVID qui a restreint l’accès aux services de santé. Cet accroissement des cas hospitalisés au fil des ans peut être la traduction d’une aggravation de la pandémie mais aussi au contraire d’une plus grande crédibilité de l’unité au sein de la communauté, grâce à une compétence affirmée et la gratuité des soins. Des investigations complémentaires sont nécessaires afin de mieux en comprendre les raisons.

Sur le plan clinique, les manifestations dominantes étaient les signes respiratoires et de la malnutrition, retrouvés chez près de 60% et 50% des patients respectivement. Les signes respiratoires sont fréquents chez ces patients, les poumons étant les organes profonds les plus extériorisés par le biais des voies respiratoires hautes. De même, la prise en charge tardive est source d’un amaigrissement important et de dénutrition.

Concernant les résultats de traitement, ce travail a mis en lumière un taux élevé de décès, en moyenne le tiers des patients soignés entre 2017 et 2021. Nos résultats corroborent ceux d’autres auteurs dans d’autres pays d’Afrique subsaharienne comme la Sierra Léone et le Nigéria.^6,10^ Chez ces patients décédés, le diagnostic retenu était de loin la TB, chez un peu moins de la moitié d’entre eux, suivie de la toxoplasmose. D’autres auteurs dans la littérature ont fait des constats similaires.^6,11^

Un point positif cependant est la réduction progressivement constatée au fil du temps des décès parmi les patients avec le VIH avancé, même si elle est encore survenue chez plus du quart des patients en 2021. La principale intervention mise en œuvre pendant la période d’étude étant la réalisation systématique du dosage des CD4, de la recherche de la TB et de la cryptococcose, en application des recommandations internationales, elle justifie très certainement la réduction de la léthalité constatée, grâce à un diagnostic moins tardif de ces infections opportunistes.^12^

Quant aux facteurs associés au décès, après prise en compte des facteurs de confusion, un âge compris entre 25–49 ans ou au-delà, ou encore la présence de signes respiratoires ou abdominaux étaient associés à un risque significativement plus élevé de survenue de cette issue défavorable. Des résultats similaires concernant l’âge n’ont pas été rapportés dans la littérature.^5,6,13^ Cependant, les patients réadmis étaient moins à risque de décès. Il est possible que les réadmissions permettent de mieux comprendre le tableau clinique du patient pour une meilleure prise en charge.

L’une des forces de cette étude est qu’elle a inclus un grand nombre de patients, près de quatre mille. Ensuite, presque tous les patients de la période d’étude ont été pris en compte, permettant de réduire les biais d’échantillonnage. Les diagnostics retenus ont été posés par des collèges de médecins, ce qui diminue les risques d’erreurs diagnostiques et les biais liés à la définition de cas. L’étude s’est aussi déroulée dans des conditions de vie réelle. Elle comporte aussi quelques limites, dont la principale limite est celle relative aux études rétrospectives, avec leurs conséquences, à savoir des erreurs d’encodage ou des données manquantes. Pour atténuer leurs effets, une procédure de contrôle qualité a été exécutée en recherchant les données manquantes et corrigeant en cas de besoin. Les données manquantes pour les variables sur les signes cliniques qui ont été incluses dans le modèle de Cox multivarié ont été recodées comme étant l’absence du signe en question.

De cette étude découlent certaines implications opérationnelles. Une attention particulière doit être accordée à ces patients avec le VIH avancé du fait de cette létalité élevée, surtout pendant les trois premières semaines d’hospitalisation qui sont les plus à risque de décès, mais aussi d’améliorer le diagnostic précoce et la prise en charge du VIH avancé au niveau santé primaire. De plus, cette étude servira à enrichir la littérature dans ce domaine au niveau national mais aussi régional. De même, il est important de continuer par rendre disponibles les intrants nécessaires aux examens de laboratoire essentiels, tels que TB-LAM, CrAg, CD4 et autres afin de les rendre systématiques à l’admission.^8^

Enfin, le développement d’activités de prise en charge ambulatoire pourrait permettre de prévenir ou de prendre précocement en charge le VIH avancé avant son arrivée à l’hôpital. En conclusion, la létalité chez les patients avec le VIH avancé a diminué entre 2017 et 2021, même si elle reste encore élevée, survenant chez plus du quart des patients pris en charge dans l’unité appuyée par MSF à l’Hôpital National de Donka. Un âge supérieur à 25 ans et la présence de signes respiratoires et abdominaux sont associés à un risque élevé de décès. Ces derniers surviennent surtout pendant les 25 premiers jours d’hospitalisation, et la TB reste la principale affection diagnostiquée parmi ces patients. Des actions visant à prévenir et à mieux prendre en charge doivent être mises en œuvre au sein de l’Unité.

### Remerciements

Cette recherche a été menée dans le cadre de l’Initiative de recherche opérationnelle et de formation structurée (SORT-IT), un partenariat mondial dirigé par le Programme spécial de recherche et de formation sur les maladies tropicales de l’Organisation mondiale de la santé (OMS/TDR). Le modèle est basé sur un cours élaboré conjointement par l’Union internationale contre la tuberculose et les maladies respiratoires (L’Union) et Médecins Sans Frontières (MSF/Doctors Without Borders). Le programme spécifique, SORT-IT, qui a donné lieu à cette publication a été organisé par MSF spécifiquement pour la recherche en langue française. Le programme a été financé par La Fondation Veuve Emile Metz-Tesch, Luxembourg. Le financeur n’a joué aucun rôle dans la conception de l’étude, la collecte et l’analyse des données, la décision de publier ou la préparation du manuscrit.

Les auteurs remercient LuxOR pour la formation et l’accompagnement tout au long de cette étude. Non remerciements vont également à la mission MSF Guinée en général, le projet VIH/TB de MSF/Guinée et SAMU en Afrique du Sud et tous les travailleurs de l’Hôpital National de Donka, Conakry, Guinée.
